# Effects of CwlM, a peptidoglycan synthesis regulator, on beta-lactam tolerance and host-pathogen interactions

**DOI:** 10.1186/s12866-025-04548-6

**Published:** 2025-12-07

**Authors:** Cátia Silveiro, Diana Mortinho, Francisco Olivença, Manoj Mandal, David Pires, Elsa Anes, Maria João Catalão

**Affiliations:** 1 Research Institute for Medicines (iMed.ULisboa), Faculty of Pharmacy, Universidade de Lisboa, 1649-003, Lisbon, Portugal; 2 Universidade Católica Portuguesa, Católica Medical School, Centre for Interdisciplinary Research in Health, Lisbon, Portugal

**Keywords:** Tuberculosis, Peptidoglycan biosynthesis, CRISPR interference, Antibiotic tolerance, Beta-lactams, Host-pathogen interactions

## Abstract

**Background:**

The emergence and spread of multidrug-resistant (MDR) strains of *Mycobacterium tuberculosis* (*Mtb*) urge the development of novel drugs and efficient therapeutic programs. A recent study aiming to uncover differential beta-lactam susceptibility phenotypes in clinical strains of *Mtb* found that the M237V substitution in *cwlM* (*Rv3915*) was associated with increased susceptibility to amoxicillin. Considering that *Mycobacterium smegmatis* (*Msm*) is a widely used surrogate model for *Mtb*, we constructed a *cwlM* knockdown mutant in *Msm* using CRISPR interference (CRISPRi) to elucidate the role of CwlM in beta-lactam susceptibility and intracellular survival.

**Results:**

Quantitative RT-PCR assays confirmed the successful repression of *cwlM*, while the phenotyping assays confirmed the essentiality of CwlM-related processes for mycobacterial growth. Collectively, the antibiotic susceptibility assays suggested that CwlM_SMEG_ may contribute to increased tolerance to meropenem and cefotaxime. Moreover, CwlM_SMEG_ was found to support *M. smegmatis* survival within THP-1-derived macrophages. To address conflicting reports regarding its predicted peptidoglycan (PG) hydrolase activity, we purified recombinant CwlM_TB_. The *Micrococcus luteus*-derived PG-based zymogram indicated that CwlM_TB_ lacks PG-hydrolytic activity, suggesting it might act as a regulator of PG biosynthesis instead.

**Conclusions:**

Our findings indicate that CwlM contributes to beta-lactam tolerance and intracellular survival, regardless of lacking detectable PG-hydrolytic activity. Overall, CwlM was found to be essential and highly vulnerable, highlighting its potential as a therapeutic target that warrants further investigation.

**Supplementary Information:**

The online version contains supplementary material available at 10.1186/s12866-025-04548-6.

## Background

Tuberculosis (TB) remains the deadliest infectious disease worldwide, having claimed 1.25 million lives in 2023 and causing over 10 million TB cases annually, a rising trend since 2021 [1]. The emergence and dissemination of multi- and extensively- drug-resistant (MDR/XDR) TB urge the identification of novel drug targets for the development of effective curative treatments [1, 2]. In this context, a structure worth exploring is the peculiar cell wall (CW) of mycobacteria, which is essential for its viability and virulence. The mycobacterial CW encompasses a distinctively cross-linked and enzymatically modified peptidoglycan (PG), a highly branched arabinogalactan (AG) polysaccharide, and characteristic long-chain mycolic acids (MA) [3, 4].

Despite its uniqueness, the mycobacterial PG is still overlooked when identifying antitubercular targets. The biosynthesis of PG starts in the cytoplasm with the generation of uridine diphosphate (UDP)-*N*-acetylglucosamine, which is then converted into UDP-*N*-acetylmuramic acid (UDP-Mur*N*Ac) by MurA and MurB [3, 4]. After, the *N*-acetylmuramic acid hydroxylase NamH catalyzes the hydroxylation of UDP-Mur*N*Ac to UDP-*N*-glycolylmuramic acid (UDP-Mur*N*Glyc) [5]. This PG modification reduces susceptibility to lysozyme and beta-lactams [5, 6] and modulates host-pathogen interactions [6–9]. The subsequent addition of L-Ala (MurC), D-Glu (MurD), *meso*-DAP (MurE) and D-Ala-D-Ala (MurF) produces the muramyl-pentapeptide, which is then anchored to the plasma membrane (PM) by MraY/MurX, generating lipid I [3, 4]. The final intracellular step of PG synthesis, the synthesis of the membrane-anchored PG precursor lipid II, is catalyzed by MurG. Both lipid I and II are then amidated at the α-carboxyl group of D-*iso*glutamate (D-*i*Glu) by the MurT/GatD amidotransferase complex [10–12]. The amidation of D-*i*Glu is essential for PG cross-linking in mycobacteria [13, 14], modulates PknB-dependent cell division (PknB is a serine/threonine protein kinase (STPK) that regulates CW biosynthesis) [15], reduces susceptibility to beta-lactams and lysozyme [6, 14], and functions as an immune evasion strategy during infection [6, 9]. Likewise, the amidation of lipid II at the ε-carboxyl group of *m*-DAP is catalyzed by AsnB [16, 17]. Apart from being essential for cell growth and L, D-transpeptidase (Ldt) activity in *Mycobacterium tuberculosis* (*Mtb*) [18], the amidation of *m*-DAP also reduces susceptibility to antibiotics and lysozyme [19, 20] as well as host immune responses. Once the MurJ flippase catalyzes the translocation of modified lipid II across the PM, the PG is polymerized by transglycosylases, penicillin-binding proteins (PBPs) and Ldts.

Beta-lactams, a broadly employed and reliable antibiotic class, could be incorporated into alternative drug-resistant TB (DR-TB) therapeutic schemes, offering improvements over the current lengthy and toxic regimens. These regimens usually exclude beta-lactams due to the intrinsic resistance of *Mtb* to these antibiotics, credited not only to a potent beta-lactamase [21] but also to the impervious layer of MA and the predominance of non-classical Ldt-catalyzed 3→3 cross-links [22, 23]. Nonetheless, several reports found that MDR-TB strains are particularly susceptible to carbapenems when combined with beta-lactamase inhibitors, such as clavulanate [24–29]. A previous study screened 172 clinical strains of *Mtb* for differential beta-lactam susceptibility phenotypes and conducted a whole genome sequencing analysis to identify cases in which the prospective addition of beta-lactams to TB therapy may yield added benefits [29]. The M237V substitution in *cwlM* (*Rv3915*), present in 50/172 isolates, was associated with increased susceptibility to amoxicillin, with and without clavulanate [29].

Being highly conserved amid mycobacteria and essential for *Mtb* survival, CwlM is predicted to be a *N*-acetylmuramoyl-L-alanine amidase, a type of PG hydrolase [30, 31]. Whereas Deng et al. [30] have shown that CwlM_TB_ possesses autolysin activity, a recent study found the function of CwlM to be regulatory rather than enzymatic [31]. CwlM is the main substrate of the STPK PknB [32]. Replicating *Mtb* produces two forms of CwlM: phosphorylated CwlM binds to FhaA, a fork head-associated domain protein, whereas non-phosphorylated membrane-associated CwlM interacts with the MurJ flippase [32]. In nutrient-replete conditions, CwlM is phosphorylated in the cytoplasm, binds to MurA, the first enzyme in PG biosynthesis, and stimulates its catalytic activity by ~30-fold [31]. In starvation or stasis, CwlM is dephosphorylated (possibly by PstP) [33], consequently downregulating PG biosynthesis and promoting antibiotic tolerance [31]. In this case, the activity of MurJ is stimulated, resulting in the accumulation of non-incorporated PG muropeptides in the periplasm [32]. Overall, CwlM simultaneously regulates the biosynthesis of PG precursors and their translocation across the PM. Therefore, the balance between both forms of CwlM, influenced by altered PknB expression or activity, is essential for cell viability [32].

Here, we implemented the dCas9_Sth1_-based CRISPRi system [34] in *Mycobacterium smegmatis*, a widely used surrogate for *Mtb*, to uncover the role of the PG hydrolase homolog, CwlM_SMEG_, in susceptibility to beta-lactams and host-pathogen interactions. Additionally, we purified recombinant CwlM_TB_ to elucidate its activity.

## Methods

### Bacterial strains, culture conditions, plasmids, cell lines, and antibiotics

A description of the bacterial strains, plasmids, and cell lines employed in this study is provided in Table 1. *Escherichia coli* (*E. coli*) strains were grown in Luria-Bertani (LB) media (Merck), overnight (ON) at 37 °C, to amplify the CRISPRi backbone PLJR962 (Addgene #115162) and for cloning. *Micrococcus luteus* (*M. luteus*) strains were also grown in Luria-Bertani (LB) media and used for the zymography assay. *M. smegmatis* strains were grown in Middlebrook 7H9 broth (BD™ Biosciences) supplemented with 0.2% glycerol (ThermoScientific), 0.05% tyloxapol (Sigma-Aldrich), and 0.5% glucose (Sigma-Aldrich). For plating procedures, mycobacteria were grown on 7H10 agar (BD™ Biosciences) with 0.5% glycerol. Bacteria were incubated at 37 °C with (liquid cultures) or devoid of (plates) shaking at 180 rpm. Media for all bacteria were supplemented with 25 µg/mL of kanamycin as appropriate. To stimulate target gene knockdown by the dCas9_Sth1_-sgRNA complex, 100 ng*/*mL of ATc were added to the cultures when necessary. RPMI-1640 media (Gibco) supplemented with 10% fetal bovine serum, 1% sodium pyruvate, 1% L-Glutamine, 1% HEPES buffer and 1% non-essential amino acids was used to grow THP-1 cells at 37 °C, with an atmosphere of 5% of CO_2_. Antibiotic stocks of amoxicillin (AMX), cefotaxime (CTX), ethambutol (EMB), isoniazid (INH), meropenem (MEM) and vancomycin (VAN) (all from Sigma-Aldrich) were prepared in purified MilliQ water to a final concentration of 1.28 mg/mL. The stocks of ATc (Sigma-Aldrich) were prepared in dimethyl sulfoxide (DMSO) cell culture grade (AppliChem) at 10 mg/mL. The stocks of potassium clavulanate (CLA) (Sigma-Aldrich) were prepared in phosphate buffer at 0.1 M, pH 6.0.

**Table 1:** Bacterial strains, plasmids, and cell lines used in this study

Bacterial strain, plasmid or cell line	Description	Reference or source
Bacteria		
*Escherichia coli*		
JM109	*recA1 endA1 gyr96 thi hsdR17 supE44 relA1 *Δ(*lac-proAB*) [F´ *traD36 proAB lac*^q^*Z*ΔM15]	Stratagene
BL21(DE3)	*fhuA2 [lon] ompT gal (λ DE3) [dcm] *Δ*hsdS λ DE3 = λ sBamHIo *Δ*EcoRI-B int::(lacI::PlacUV5::T7 gene1) i21 *Δ*nin5*	New England Biolabs
*Mycobacterium smegmatis*		
mc^2^ 155 (WT)	High-transformation-efficiency mutant of *M. smegmatis* ATCC 607	[35]
Plasmids		
PLJR962 (8881 bp)	Integrative plasmid Sth1 *dcas9* Tet^R^ and Kan^R^ L5 Int *attP* for *M. smegmatis*	[34]
Cell lines		
THP-1	Human-derived monocyte cell line	ATCC, TIB-202

### Cloning of the CRISPRi plasmid and preparation of the *cwlM* knockdown mutant

The cloning techniques were carried out as formerly described [6, 34, 36]. The non-template strand of the gene of interest (GOI), *cwlM* (*MSMEG_6935*) was targeted, to produce efficient silencing. Since *cwlM* is putatively essential for *Mtb* survival [30], the template strand of *cwlM* was searched for a PAM associated with medium-to-low repression and accordingly guided sgRNA design (Table 2). The CRISPRi backbone PLJR962 was amplified in *E. coli* JM109 and digested as previously described [6]. For sgRNA cloning, two complementary primers were designed (PFwd_sgRNA_cwlM: 5’ **-** GGGAACCCGCCGGTGACGCGTGAGC − 3’; PRv_sgRNA_cwlM: 5’ - AAACGCTCACGCGTCACCGGCGGGT − 3’), synthesized (Eurofins Genomics), annealed, and ligated to the backbone vector with T4 DNA ligase (400 U/µL; NEB). To obtain the interference plasmid, *E. coli* competent cells were transformed with the ligation mixture by double heat-shock, and the resulting recombinant DNA was purified with the NZYMiniprep kit (NZYTech). *M. smegmatis* competent cells were electroporated with 300 ng of recombinant DNA in the presence of 10% glycerol to generate the *cwlM* knockdown mutant (KDM) [6, 37].

**Table 2:** sgRNAs used to target *cwlM* in *M. smegmatis*

Target gene	*cwlM*
Oligo	sgRNA1
PAM sequence and strength ^a^	5 ‘- CGAGGAA − 3’ (PAM 14)
Gene Location 5’−3’ (bp)	467
Base-pairing region of the sgRNA (the 12 bp seed region is underlined)	5’ - ACCCGCCGGTGACGCGTGAGC − 3’
BLASTed sequence in the genome of *M. smegmatis* (seed region + PAM)	5’ - TGACGCGTGAGCCGAGGAA − 3’
Fold knockdown efficiency ^b^	2.5

^a^PAM strength was described in accordance with the table of functional PAMs in vivo for dCas9_Sth1_-mediated targeting in mycobacteria defined by Rock et al.

^b^mRNA knockdown caused by the electroporation of the indicated sgRNA in the *M. smegmatis* WT strain was measured by qRT-PCR after ATc induction. Fold knockdown efficiency is relative to a non-induced control plasmid with the same sgRNA

### RNA extraction and quantitative reverse-transcription PCR (qRT-PCR)

The collection of cultures for total RNA extraction was performed as previously described [6]. Briefly, induction with 100 ng/mL of ATc occurred at an optical density at 600 nm (OD_600_) of about 0.1 and the cultures were then allowed to grow for 6 h at 37 °C. The cells were harvested, resuspended in 500 µL of RNAprotect Bacteria Reagent (Qiagen) and stored at −80 °C. Lysis was carried out with 350 µL of buffer NR (NZYTech) and 3.5 µL of beta-mercaptoethanol (Merck), followed by mechanical disruption in a BeadBug™ (Benchmark Scientific). Bead-beating occurred for 4 cycles (each with 1 min) at maximum speed, with 1 min incubations on ice between each cycle. After, the lysate was transferred into the NZYSpin Homogenization column (NZYTech), and the following steps were performed according to the instructions provided by the NZYTotal RNA Isolation Kit. RNA was eluted with warm DEPC water (Invitrogen) and rigorously treated with TurboDNase (Invitrogen) to prevent genomic DNA contamination. The obtained RNA was quantified using Nanodrop™ and the lack of DNA contamination was corroborated by PCR on the housekeeping gene *sigA*, followed by gel electrophoresis analysis [6].

Reverse transcription reactions were performed using 100 ng of purified RNA as template, following the instructions of the NZY First-Strand cDNA Synthesis kit (NZYTech). Afterwards, cDNA was quantified using the NZYSupreme qPCR Green Master Mix (2x), ROX (NZYTech) on a QuantStudio™ 7 Flex Real-Time PCR System (Applied Biosystems). The 40-cycle qPCR reaction ensued in this way: 95 °C for 2 min, then 95 °C for 5 s and 60 °C for 30 s. The employed primers (Eurofins Genomics; Table 3) were designed to amplify a specific product between 100 and 200 bps, and their amplification efficiency was assessed by calibration curves and proven to be 80–110%. For every run, a minimum of two technical replicates and one negative control were utilized, and the mean C_T_ was calculated. Data were analyzed using the ΔΔC_T_ method with *sigA* (*MSMEG_2758*) as a reference gene and *M. smegmatis* WT as the calibrator sample [34, 36, 38]. Two independent measurements were used to quantify the target mRNA sequences [6, 39].

**Table 3:** Primers used to quantify the mRNA expression of target genes by qRT-PCR in *M. smegmatis*

qRT-PCR primers^a^							
Target gene	Primer sequences		Length (bp)	%GC	Tm^b^ (°C)	Product size (bp)	AE^c^ (%)
*sigA* (*MSMEG_2758*)	PFwd	5’ - ACACCGACCTGGAACTCG − 3’	18	61	58	152	80
	PRv	5’ - GACGCCTTGTCCTTCTCG − 3’	18	61	58		
*cwlM* (*MSMEG_6935*)	PFwd	5’ - GGTAAGCGGGTCATCATCG − 3’	19	58	59	104	108
	PRv	5’ - GGTCCCACAGGATGTCG − 3’	17	65	58		

^a^qRT-PCR primers were designed to avoid primer secondary structures and sequence repeats, have at least 50% of GC content, and have a Tm between 57 and 63 °C

^b^Calculated with the Eurofins Genomics Tm formula

^c^AE designates the amplification efficiency calculated for each pair of primers in a single-run qPCR amplification of the calibrator sample (*M. smegmatis* WT), performed in triplicates

### Phenotyping

To evaluate growth differences following CRISPRi induction, spotting assays were performed as previously described [6]. Briefly, cultures of *M. smegmatis* were allowed to reach the log phase of growth, normalized to a theoretical OD_600_ of 0.001, and serially diluted. A 5 µL aliquot from each dilution was plated on agar media, which could be devoid of antibiotics or contain meropenem (MEM; 0.25, 0.5, and 1 µg/mL) or cefotaxime (CTX; 8, 16, and 32 µg/mL) at different concentrations, as indicated in brackets. The plating of each bacterial suspension occurred in both induced and non-induced conditions, following incubation at 37 °C for 2 days. At least three independent experiments were performed.

### Minimum inhibitory concentration (MIC) determination

To uncover differences in antibiotic susceptibility, the MICs of three beta-lactams (AMX, CTX and MEM), with and without the beta-lactamase inhibitor clavulanate, as well as one glycopeptide (vancomycin) and two first-line antimycobacterial agents (INH and EMB), were determined as formerly described [6]. The MIC was annotated as the lowest concentration of antibiotic capable of preventing visible bacterial growth. The median MIC values were calculated from three independent experiments.

### Disk diffusion assays

To determine susceptibility differences on solid media following CRISPRi induction, disk diffusion assays were performed with MEM or AMX + CLA, as described previously [21, 40]. Briefly, the cultures of *M. smegmatis* were grown to log phase, normalized to a theoretical OD_600_ of 0.05, and uniformly distributed over the 7H10 (+ ATc) plates using a pour-plate technique. Subsequently, antibiotics were prepared at the indicated test concentrations (MEM: 256 and 512 µg/mL; AMX: 512 and 1024 µg/mL; CLA: 2.5 µg/mL). Then, 20 µL of each antibiotic dilution was added to 9 mm filter paper disks (Filterlab), which were allowed to dry and placed onto the mycobacterial lawn using a sterile needle. The plates were then incubated at 37 °C for 2 days, after which photographs were captured on an iBright FL1500 Imaging System (Invitrogen) and the diameter of the zone of inhibition (halo) was measured (in mm) using the ImageJ software. Three independent experiments were performed.

### THP-1 infection assays and intracellular survival evaluation

To explore the role of CwlM in intracellular survival, THP-1-derived macrophages were infected with control and mutant bacteria. THP-1 monocytes were passaged every 3–4 days, being kept at a density above 2 × 10^5^ cells/mL. When necessary, the cells were seeded in 48-well flat-bottom tissue culture plates at a density of 1.5 × 10^5^ cells/well and differentiated into macrophages with 20 nM of phorbol 12-myristate 13-acetate (PMA; Sigma-Aldrich) for 24 h [41]. On the day of THP-1 seeding, the bacterial cultures were diluted to an OD_600_ of about 0.007 and incubated at 37 °C with shaking till an OD_600_ of approximately 0.1 was reached. At that point (~ 24 h before infection), the cultures were incubated with or without 100 ng/mL of ATc.

Approximately 24 h before infection, the cell media containing PMA was removed and replenished with prewarmed supplemented RPMI media. On the day of infection, bacterial cultures were centrifuged at 3,000 x g for 5 min and washed with 5 mL of PBS 1 X (Gibco) [42]. The resultant pellets were then resuspended in supplemented RPMI media, subjected to an ultrasonic bath for 5 min, and centrifuged at 500 x g for 1 min to obtain a single-cell suspension [43]. After discarding the cell media from the seeding plates, THP-1-derived macrophages were infected with mycobacteria at a multiplicity of infection (MOI) of 5 and, incubated at 37 °C with 5% CO_2_ for several timepoints (1 h; 4 h; 24 h) [6, 42]. After one hour of internalization, the infection media was discarded, macrophages were washed thrice with prewarmed PBS 1 X, and resuspended in supplemented RPMI media containing 50 µg/mL gentamicin to kill extracellular bacteria and 100 ng/mL of ATc, when suitable. Following distinct pre-determined incubation times (T_1_, T_4_, T_24_), the media was discarded, the cells were washed thrice with PBS 1 X and, incubated at 37 °C with a 0.05% IGEPAL solution for 15 min to induce lysis [43]. After, the lysates were serially diluted in sterile water and plated onto 7H10 plates, which were incubated for 2 days at 37 °C with 5% CO_2_ [42]. Using an inverted microscope (Nikon TMS Inverted Phase Contrast Microscope), the colony-forming units (CFU) of three independent biological replicates, each counting three technical replicates, were counted.

### Cloning of recombinant CwlM_TB_

To overexpress and characterize the CwlM_TB_ protein, the *cwlM* gene (*Rv3915*) was cloned in frame with a C-terminal polyhistidine (His6) tag into the pET29b backbone, as previously described [40, 44]. The oligonucleotides PFwd_*cwlM* (5’ – GACCATATGCCGAGTCCGCGCCGCG – 3’) and PRv_*cwlM* (5’ – GCCCTCGAGAGAACCGCCGAGTCTACC – 3’) were used to amplify the *cwlM* gene from 30 ng of *Mtb* H37Rv genomic DNA using 0.025 U/µL of Supreme NZYProof DNAPolymerase (NZYTech). The PCR was conducted on a thermocycler (Life Technology - SimpliAmp), with the following PCR program: 96 °C for 4 min, 1 cycle; 96 °C for 30 s, 63 °C for 30 s and 72 °C for 40 s, 30 cycles; 72 °C for 10 min, 1 cycle. The 1234 bp PCR product was confirmed by 0.8% agarose gel electrophoresis and visualized using the iBright™ FL1500. Afterwards, the PCR reaction was scaled up, the PCR product was purified using the NZYGelpure kit (NZYTech) and, 800 ng of purified PCR product and 600 ng of the pET29b plasmid were then digested at 37 °C for 3 h with 2.5 µL of FastDigest Xho*I* and Nde*I* (ThermoScientific) in a final volume of 80 µL. The reaction products were purified using the NZYGelpure kit (NZYTech) and separated by a 0.8% agarose gel electrophoresis, followed by visualization on iBright™ FL1500. The digested fragment was then ligated to 50 ng of digested pET29b with 80 U of T4 DNA ligase (400 U/µL; NEB) using a vector-to-insert ratio of 1:5, at 22 °C for 1 h in a ThermoMixer C (Eppendorf). Afterwards, *E. coli* JM109 chemically competent cells were transformed with 5 µL of ligation mixture using a double heat-shock approach, and the recombinant DNA was purified according to the instructions of the NZYMiniprep kit (NZYTech). The obtained recombinant plasmid was then quantified, evaluated for purity, and sequenced using primers that specifically amplify the T7 RNA polymerase promoter PFwd_T7 (5’ – TAATACGACTCACTATAGGG – 3’) and terminator PRv_T7 (5’ – CTAGTTATTGCTCAGCGG – 3’).

### Expression of recombinant CwlM_TB_ in *E. coli* BL21(DE3) and SDS-PAGE analysis

*E. coli* BL21 (DE3) chemically competent cells were transformed with 300 ng of the pET29b:*cwlM* construct using a double-heat shock procedure [40]. Inoculums from *E. coli* BL21(DE3):pET29b:*cwlM* transformants were prepared and incubated at 37 °C with shaking until the OD_600_ was about 0.5. Then, cultures were induced with 1 µM of IPTG (NZYTech), incubated in two distinct conditions, at 37 °C for 3 h and at 16 °C overnight, collected by centrifugation, and frozen at −20 °C [40]. The next day, pellets were resuspended in a lysis buffer solution [(50 mM Tris, 300 mM NaCl), supplemented with 1% of a cocktail of protease inhibitors (Calbiochem), 10 µg/mL DNAse, 10% glycerol and 0.1% Triton X-100], sonicated twice for 60 s (0.5 cycles, 100% amplitude, pulse on, on ice), centrifuged at 8,000 x g, 4 °C for 20 min, separated from the supernatant, and stored at −20 °C. SDS-PAGE analysis of the obtained pellets and supernatants was performed in a Mini-PROTEAN^®^ Tetra Cell to verify the presence of the protein of interest, according to the standard protocol Hand Casting Polyacrylamide Gels by Bio-Rad [40]. To prepare the ready-to-load samples, 6 µL of loading dye (5 X SDS-PAGE Sample Loading Buffer; NZYTech) were combined with 24 µL of the supernatants. Pellets were resuspended in 24 µL of running buffer 1 × (50 mM Tris, 500 mM glycine (pH 8.3), 0.1% w/v SDS), and then mixed with 6 µL of loading dye (5 X SDS-PAGE Sample Loading Buffer). Afterwards, the samples were heated at 100 °C for 5 min and iced for 1 min. An amount of 20 µL of each sample was applied to the gel, the stacking gel ran at 80 V and the resolving gel ran at 120 V. After the run, the gel was stained with BlueSafe (NZYTech) for approximately 30 min. Based on the comparison of soluble protein yields under both induction conditions, we chose to induce 2 L of culture at 16 °C overnight [40].

### Purification of CwlM_TB_

After induction, the scaled-up 2 L culture was collected by centrifugation, and the resulting pellet of *E. coli* BL21(DE3) cells expressing CwlM_TB_ was thawed on ice and resuspended in 40 mL of lysis buffer [40]. Lysis was achieved by sonication in 10 cycles of 30 s, 50% amplitude, interpolated by 40 s rest periods. Afterwards, the lysate was centrifuged (Centrifuge 5810 - Eppendorf) at 11,200 x g, 4 °C, for 45 min and the supernatant was filtered using 0.45 µM and 0.2 µM filters (GE Healthcare). The soluble extract was then loaded in ÄKTAprime plus and purification of the His_6_-tagged CwlM_TB_ protein was carried out with a HisTrap FF Crude 1 mL Column (Cytiva), according to the instructions provided by the manufacturer. Washing buffer (50 mM Tris, 300 mM NaCl, 40 mM Imidazole, 1 mM DTT, pH 8.0), elution buffer (50 mM Tris, 300 mM NaCl, 300 mM Imidazole, 1 mM DTT, pH 8.0), MilliQ water, and 20% ethanol were also loaded in the system when appropriate. The bound CwlM_TB_ protein was eluted in the presence of imidazole, a competitive agent of His_6_-tagged proteins [45]. After elution, all fractions were collected, pooled, and the buffer was exchanged by overnight dialysis at 4 °C with magnetic stirring, using dialysis buffer (20 mM Tris, 150 mM NaCl, 25% glycerol, 1 mM DTT) [40]. Next, the protein sample was centrifuged at 10,000 x g, 4 °C, for 10 min and filtered using 0.2 µM filters. The purified CwlM_TB_ protein sample was subsequently quantified by the Bradford method, using bovine serum albumin (BSA, 10 µg/mL) (Sigma-Aldrich) as a standard [40].

### Zymography study to characterize the enzymatic activity of the CwlM_TB_ protein

To uncover whether the CwlM_TB_ protein has PG-hydrolytic activity, a zymography assay was carried out against PG obtained from *M. luteus*. First, the bacteria were grown at 37 °C for two days until exhaustion, centrifuged at 4,000 x g, 4 °C, for 10 min, and the pellet was washed once with MilliQ water. The pellet was then resuspended in MilliQ water, autoclaved, and harvested by centrifugation at 12,000 x g, 4 °C for 20 min. After discarding the supernatant, the pellet was dried at 37 °C for 24 h, weighed, and a 2% solution of *M. luteus* PG in MilliQ water was prepared and stored at −20 °C. The zymogram gel was prepared according to Bio-Rad instructions (12% running gel and 5% stacking gel) and the 2% solution of *M. luteus* PG was added to the appropriate volume of water to achieve a final concentration of 0.2% [40]. An equivalent SDS-PAGE gel without *M. luteus* PG was also prepared to track the run. Loaded samples comprised 5 µg of BSA (negative control, NZYTech), 5–10 µg of CwlM_TB_ and 1–2 µg of Lysozyme (Lys, positive control, Sigma-Aldrich) in sample buffer (62.5 mM Tris-HCl, pH 6.8, 2% SDS, 5% β-mercaptoethanol, 20% glycerol, 0.01% bromophenol blue). Samples were heated at 100 °C for 5 min and iced for 1 min. A small volume (4 µL) of the NZYColour Protein Marker II (NZYTech) and 20 µL of each sample were loaded onto the gels, which were subsequently run and stained with BlueSafe. The zymogram gel was washed in distilled water for 30 min at RT, and then incubated in renaturation buffer (5% Triton X-100, 25 mM Tris-HCl, pH 7.5) at 37 °C, for 16 h, with shaking at 120 rpm. After renaturation, the gel was washed twice for 15 min, tinted with a staining solution (0.2% methylene blue, 0.01% KOH) for 30 min, and repeatedly de-stained with distilled water until the protein ladder was visible 40]. Gel visualization was performed using the iBright™ FL1500. The zymography assay was only performed once.

### Statistical analysis

GraphPad (version 8.4.3.686) was used for statistical analysis and data are here presented as mean ± SEM, unless otherwise specified. The qRT-PCR fold expression values were tested for normality using Q-Q plots, whereas the CFU counts were analyzed through the Shapiro-Wilk Test. Multiple group comparisons were accomplished using ordinary one-way ANOVA and pairwise comparisons of selected groups were evaluated via the Holm-Sidak test. All the prerequisites of the tests were verified.

## Results

### Construction and characterization of the *cwlM* knockdown mutant

**Fig. 1:**
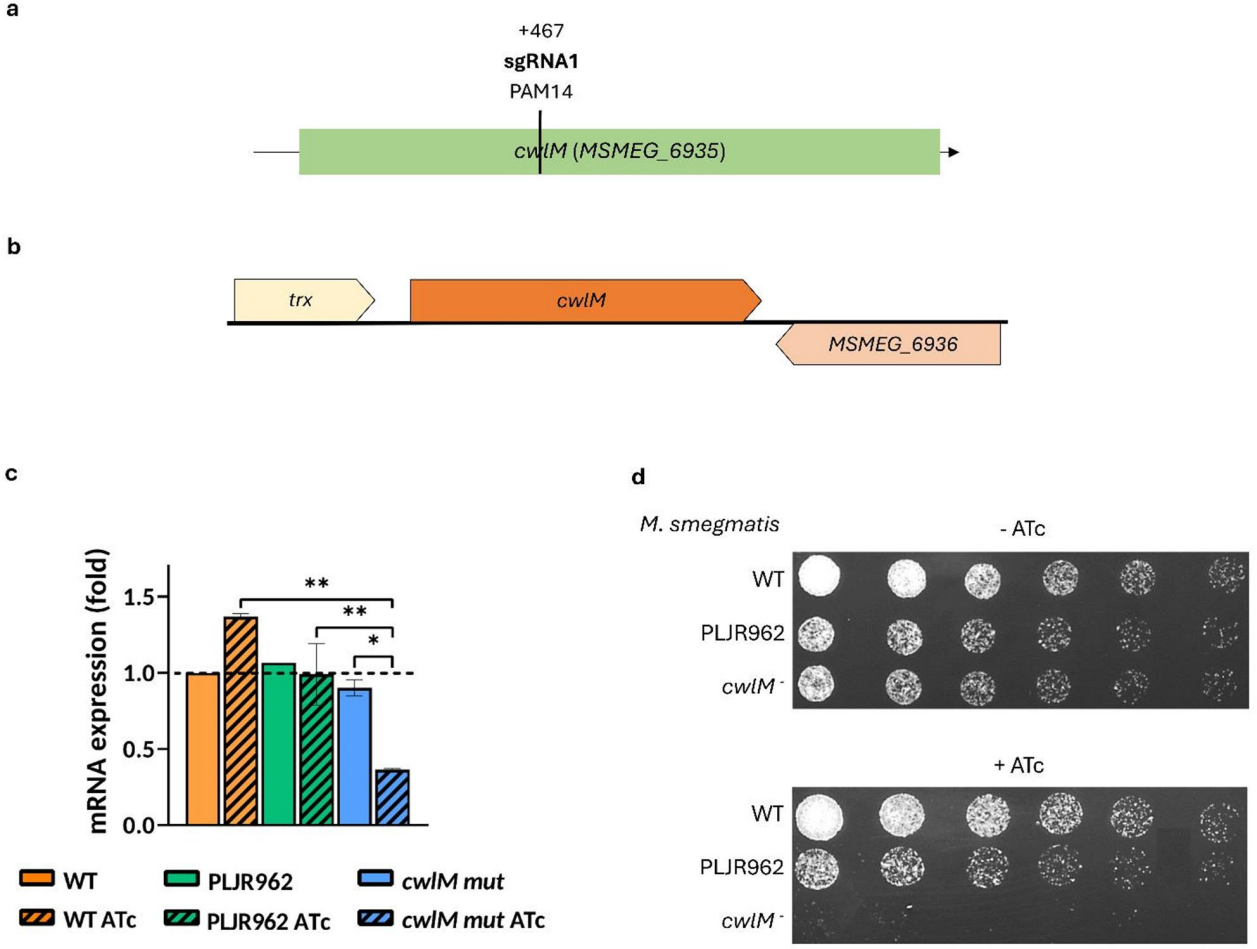
CRISPRi-mediated silencing of the *cwlM* gene in *M. smegmatis* and resulting phenotypical and mRNA expression differences.** a** sgRNA-mediated repression of the *cwlM* gene in *M. smegmatis*, detailing target site (+ 467 bps) and the employed PAM strength (PAM14). **b** The local context of the *cwlM* gene in *M. smegmatis*. The *cwlM* gene is non-operonic, being preceded by the *trx* gene and succeeded by *MSMEG_6936*, which is transcribed in the opposite direction. **c** Relative gene expression of *cwlM* normalized to *sigA*, with (stripped bars) and without (smooth bars) 100 ng/mL of ATc treatment for 6 h, evaluated through qRT-PCR assays (*n* = 2). PLJR962 is a non-targeting empty vector control. The mRNA expression levels of the calibrator sample (*M. smegmatis* WT) are shown as dashed lines. The error bars represent the standard error of the mean (SEM). Comparisons between multiple groups were made using one-way ANOVA, with significance levels: * *P* < 0.05; ** *P* < 0.01. **d** Spotting dilution assays of the negative controls (*M. smegmatis* WT, PLJR962) and of the *cwlM* knockdown mutant (*n* = 3). The first spots were normalized to an OD_600_ of 0.001, and the subsequent spots correspond to two-fold serial dilutions

The dCas9_Sth1_-based CRISPRi system [34] was implemented to construct a *cwlM* KDM in *M. smegmatis*. To produce an efficient repression of *cwlM* (*MSMEG_6935*), the non-template strand was targeted. The efficiency of CRISPRi-mediated knockdown is influenced by several factors, including PAM strength, sgRNA-DNA_target_ complementarity, target site, and GC content [6, 34]. The strength of the employed PAM is usually associated with the produced knockdown level and the resultant growth inhibition phenotypes [6]. As the repression of essential genes, such as *cwlM* [30], might cause cell death when mediated by strong PAMs, the first half of the template strand of *clwM* was searched for a medium-to-low strength PAM (Fig. 1a). Choosing a target site in the first half of the GOI is important to ensure that the produced mRNAs do not originate truncated proteins retaining some degree of activity. Although polar effects are common with CRISPRi [34, 38, 39], *cwlM* repression is unlikely to affect the transcription of neighbouring genes, as it is a non-operonic gene (Fig. 1b). Before mycobacterial transformation, the *cwlM*-targeting CRISPRi plasmid was verified by Sanger Sequencing (Additional file 1: Figure S1).

Following this, the normalized relative gene expression of *cwlM* was determined for the controls *M. smegmatis* WT and PLJR962 and the *cwlM* KDM, with and without inducer, at 6 h post-induction. The results show that the *cwlM* gene was successfully silenced (Fig. 1c). The sgRNA1-mediated knockdown of *cwlM* produced a very significant decrease in the target mRNA expression levels compared to WT ATc (3.8-fold; *P* = 0.0015) and to PLJR962 ATc (2.5-fold; *P* = 0.0029). The repression of *cwlM* was also determined as significant relative to the respective non-induced control (2.7-fold; *P* = 0.02) (Fig. 1c).

Afterwards, spotting dilution assays were performed to facilitate the phenotypical characterization of the *cwlM* KDM. The growth of negative controls *M. smegmatis* WT and PLJR962 was not affected by ATc treatment (Fig. 1d). Conversely, the *cwlM* KDM suffered a severe growth defect in the presence of inducer (Fig. 1d).

### Influence of CwlM depletion on antibiotic susceptibility

**Fig. 2:**
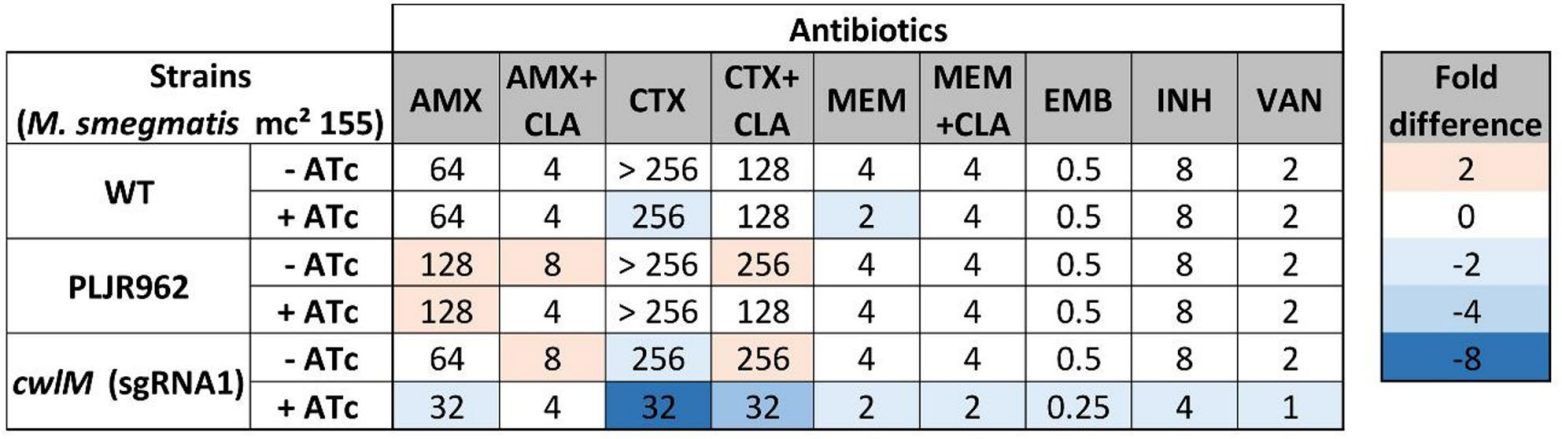
Antibiotic susceptibility assays of the negative controls and the *cwlM* knockdown mutant. Heatmap of the fold differences in the minimum inhibitory concentrations (MICs; in µg/mL) between the *cwlM* knockdown mutant, the non-targeting control PLJR962 and *M. smegmatis* WT, with and without 100 ng/mL of ATc. The red shades depict an increase in the MIC while the blue shades depict a decrease in the MIC, when compared to the WT strain. AMX, amoxicillin; CLA, clavulanate; CTX, cefotaxime; EMB, ethambutol; INH, isoniazid; MEM, meropenem; VAN, vancomycin

To uncover whether the depletion of CwlM provokes any considerable changes in antibiotic susceptibility, the MICs of EMB, INH, VAN and of three beta-lactams (AMX, CTX and MEM) in the presence or absence of clavulanate, were determined against the control strains and the *cwlM* KDM (Fig. 2).

As expected, *M. smegmatis* WT and PLJR962 did not suffer extensive changes in susceptibility to the tested antibiotics, even with inducer [40]. Notably, the knockdown of *cwlM* promoted subtle but reproducible MIC reductions to all tested antibiotics. Consistent with commonly accepted thresholds for microdilution susceptibility studies, 2-fold MIC differences were attributed to technical variability; therefore, while we recognize that consistent 2-fold MIC reductions could suggest an overall trend toward increased susceptibility, only MIC differences of ≥ 4-fold were considered biologically meaningful. The depletion of CwlM did not seem to substantially affect the susceptibility of mycobacteria to first-line agents EMB and INH, AMX (+ CLA), MEM (+ CLA) and to the PG cross-linking inhibitor VAN [40]. Conversely, the repression of *cwlM* promoted a noteworthy increase in susceptibility (8-fold) to CTX (+ CLA) [40]. The addition of CLA to AMX and CTX produced 16-fold and 2-fold MIC reductions, respectively. On the other hand, the addition of CLA to MEM did not provoke any MIC changes.

### CwlM knockdown promotes increased susceptibility to beta-lactams on solid media

**Fig. 3:**
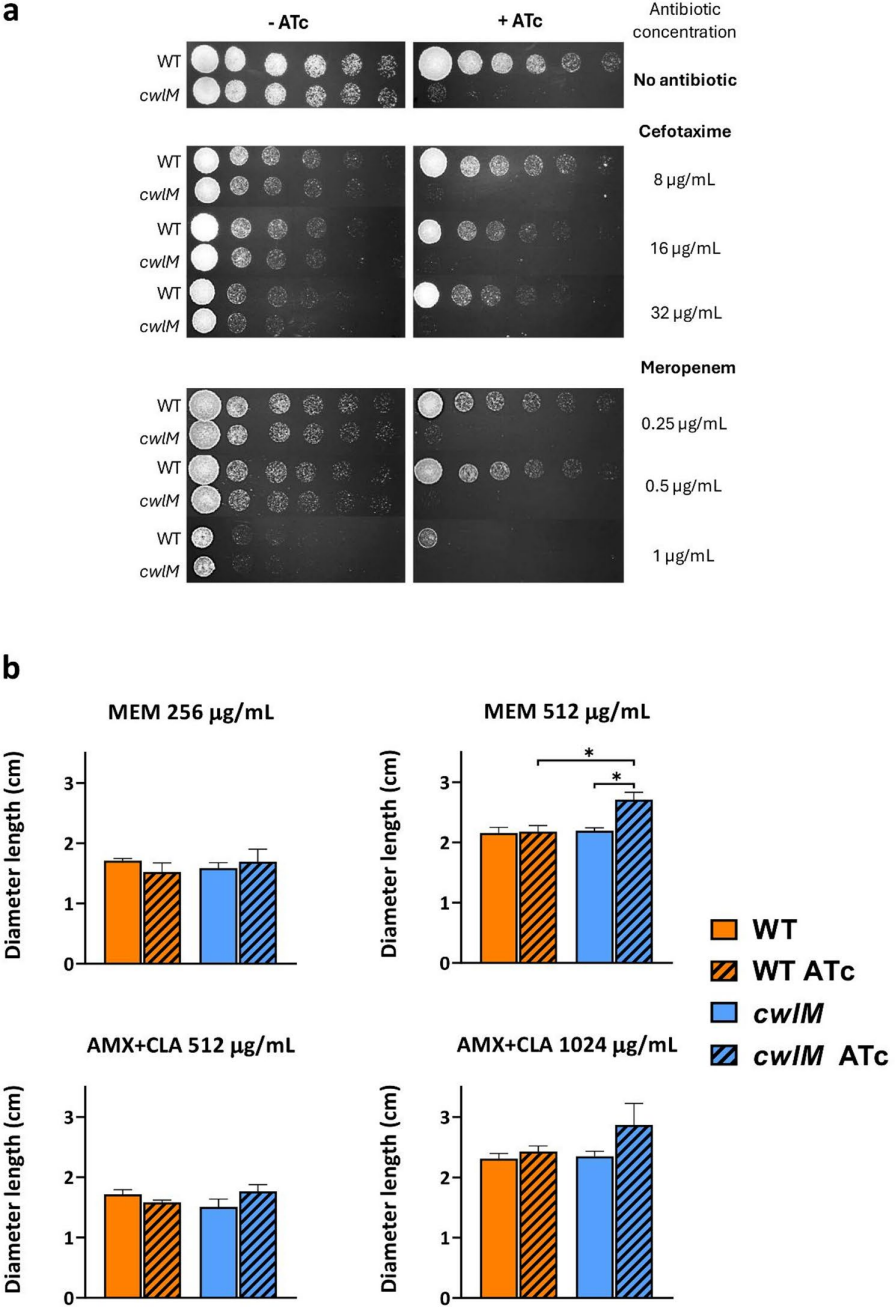
Susceptibility assays of the negative controls and the *cwlM* knockdown mutant on solid media.** a** Spotting dilution assays of the negative control strain *M. smegmatis* WT and the *cwlM* knockdown mutant, with and without 100 ng/mL of ATc, without any antibiotics and in presence of varying concentrations of cefotaxime (8, 16, and 32 µg/mL) and of meropenem (0.25, 0.5, and 1 µg/mL). **b** Average diameter of the inhibition halo (in cm) obtained for amoxicillin supplemented with clavulanate (AMX + CLA) and meropenem (MEM) antibiotic disk assays performed with the negative control *M. smegmatis* WT and the *cwlM* knockdown mutant, with (striped bars) and without (smooth bars) 100 ng/mL of ATc (*n* = 3). The diameter of the inhibition halo was measured using ImageJ. The error bars correspond to the standard deviation. Multiple group comparisons were calculated using ordinary one-way ANOVA followed by the Holm-Sidak’s test (* *P* < 0.05)

To validate these observations, spotting dilution assays of the control *M. smegmatis* WT and the *cwlM* KDM were performed in the presence of varying concentrations of CTX and MEM, with and without inducer (Fig. 3a). As formerly demonstrated, the *cwlM* KDM suffered a severe growth defect in the presence of 100 ng/mL of inducer. The growth of *M. smegmatis* WT remained mostly unaffected regardless of the concentration of antibiotics tested. Nevertheless, a marked growth defect was observed with 1 µg/mL of MEM [40]. Moreover, the growth profile of the *cwlM* KDM in non-induced conditions was very similar to that of the WT strain, with both antibiotics. On the other hand, the induced *cwlM* KDM presented a slight growth defect with both MEM and CTX when compared to the control strain [40]. In fact, the induced *cwlM* mutant tended to form more colonies at lower antibiotic concentrations, whereas a reduced number of colonies were observed at higher antibiotic concentrations.

To further consolidate these findings, AMX + CLA and MEM disk diffusion assays were performed for *M. smegmatis* WT and the *cwlM* KDM, with and devoid of inducer (Fig. 3b). As follows, the concentrations of antibiotics used to produce a visible halo effect had to be optimized. For AMX + CLA, this concentration was 8-fold greater than the MIC determined in liquid media and for MEM, it was 64-fold the MIC. CTX was excluded from this assay due to the necessity of preparing a highly concentrated stock solution defying solubility limitations. Unlike with 256 µg/mL of MEM, the induced *cwlM* KDM displayed a significant increase in susceptibility to 512 µg/mL of the carbapenem when compared to the induced WT strain (1.25-fold; *P* = 0.012) and to the respective non-induced control (1.24-fold; *P* = 0.015) [40]. No significant differences in the halo diameter were observed between WT ATc and the induced *cwlM* KDM at 512 µg/mL of AMX + CLA, while the induced KDM displayed a modest, non-significant increase in susceptibility to the penicillin at 1024 µg/mL [40].

### Impact of CwlM depletion in the killing of mycobacteria within THP-1-derived macrophages

To uncover whether CwlM contributes to survival within host macrophages, THP-1-derived macrophages were infected with the control strains and the *cwlM* KDM at a MOI of 5. The intracellular survival (in CFU/mL) was then assessed following cell lysis at 1 h, 4 h, and 24 h post-infection. No considerable differences in in vitro growth kinetics were observed among the study strains prior to infection; OD_600_ values differed by less than 0.23 across all strains (Additional file 1: Table S1).

**Fig. 4:**
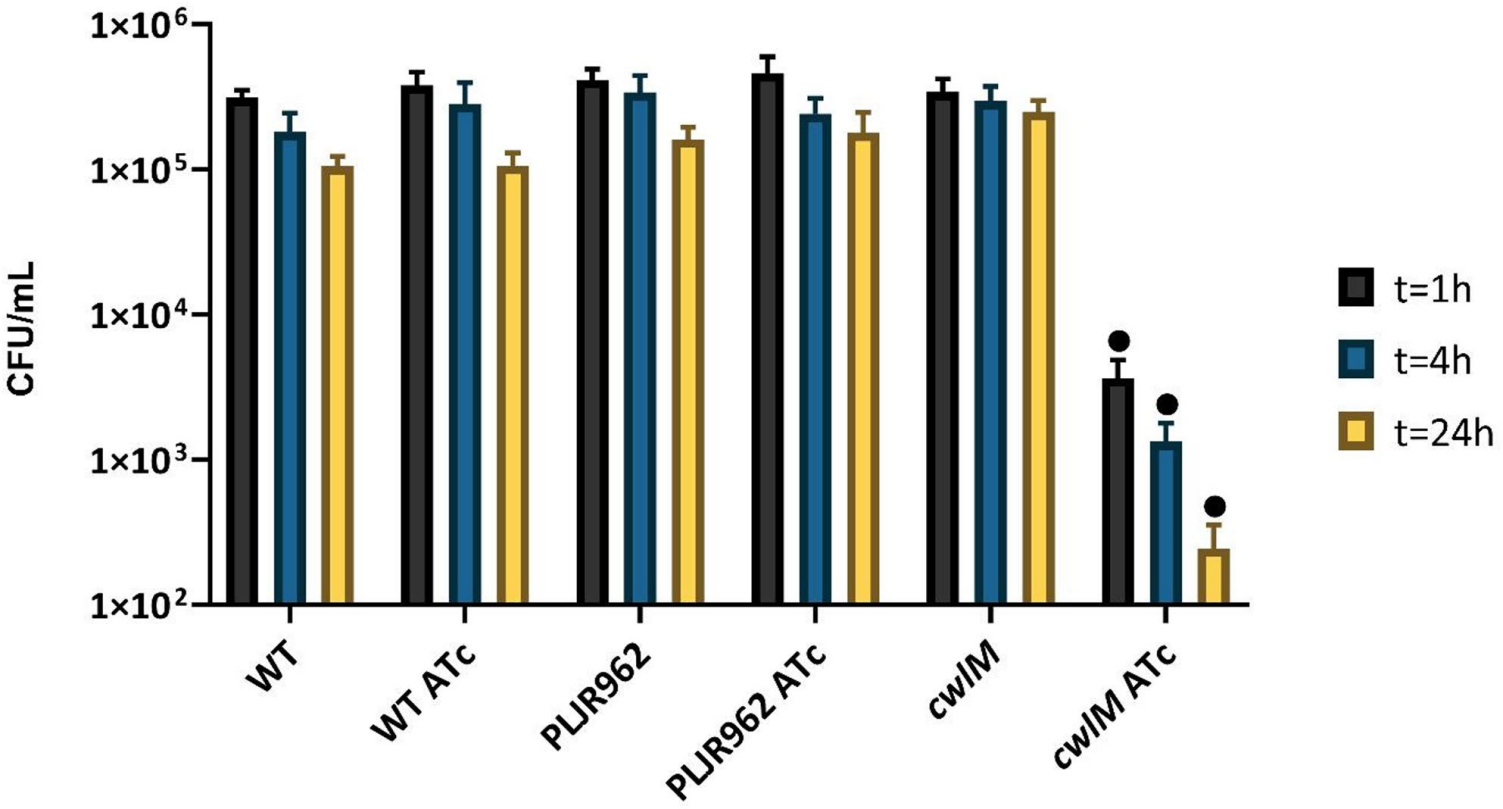
Logarithmic representation of the mean survival, in CFU/mL, of bacteria recovered from infected and disrupted PMA-differentiated THP-1-derived macrophages (*n* = 3). The disruption of macrophages occurred at 1 h, 4 h, and 24 h post-infection. Comparative effect with and without 100 ng/mL of inducer (ATc). Error bars represent the standard error of the mean (SEM). Multiple comparisons were calculated using one-way ANOVA (• *P* < 0.001 relatively to all pre-selected control strains)

As expected, the survival of mycobacteria within THP-1-derived macrophages reduced over time [6]. Moreover, the mean intracellular survival of the negative controls *M. smegmatis* WT and PLJR962 was not significantly affected by the presence of inducer. The knockdown of *cwlM* induced a very significant reduction in intracellular survival at all time points (*P* < 0.001, when compared to *M. smegmatis* WT ATc, PLJR962 ATc, and the corresponding non-induced control) (Fig. 4). Notably, the survival of the induced *cwlM* knockdown mutant within host macrophages consistently decreased over time, being significantly lower than that of *M. smegmatis* PLJR962 ATc (3.1-fold; *P* = 0.024) and of the respective non-induced control (6.3-fold; *P* < 0.001) at T_24_ (Additional file 1: Figure S2). These results suggest that the depletion of CwlM promotes a faster clearance of intracellular mycobacteria, possibly due to increased macrophage-mediated immune recognition of mycobacteria.

### Cloning, expression and purification of Recombinant CwlM_TB_

To produce and characterize the CwlM_TB_ protein, the pET system was employed to clone the *cwlM* gene (*Rv3915*) in frame with a C-terminal polyhistidine (His_6_) tag [40]. Following the verification of construct integrity through Sanger sequencing analysis (Additional file 1: Figure S3), recombinant *E. coli* BL21(DE3) cells were used to overexpress *cwlM* [40]. Induction with 1 µM of IPTG was tested under distinct conditions (37 °C for 3 h or at 16 °C overnight) to optimize overexpression of the CwlM_TB_ protein (43.9 kDa) in pET29b. SDS-PAGE analysis of the resulting fractions showed a discernible band with the expected size of 43.9 kDa in the fourth lane (CwlM at 16 °C overnight), compared to the non-induced (N.I) control (Additional file 1: Figure S4a) [40]. Overall, these results demonstrate that CwlM_TB_ is only effectively produced when the cells are grown at 16 °C overnight. Based on these findings, a scale-up culture of 2 L of *E. coli* BL21(DE3):pET-29b:*cwlM* was induced under optimal expression conditions. SDS-PAGE analysis of the resulting fractions confirmed the successful production of CwlM_TB_, with a visible band at 43.9 kDa in the third lane, which was absent in the N.I. control (Additional file 1: Figure S4b) [40].

**Fig. 5:**
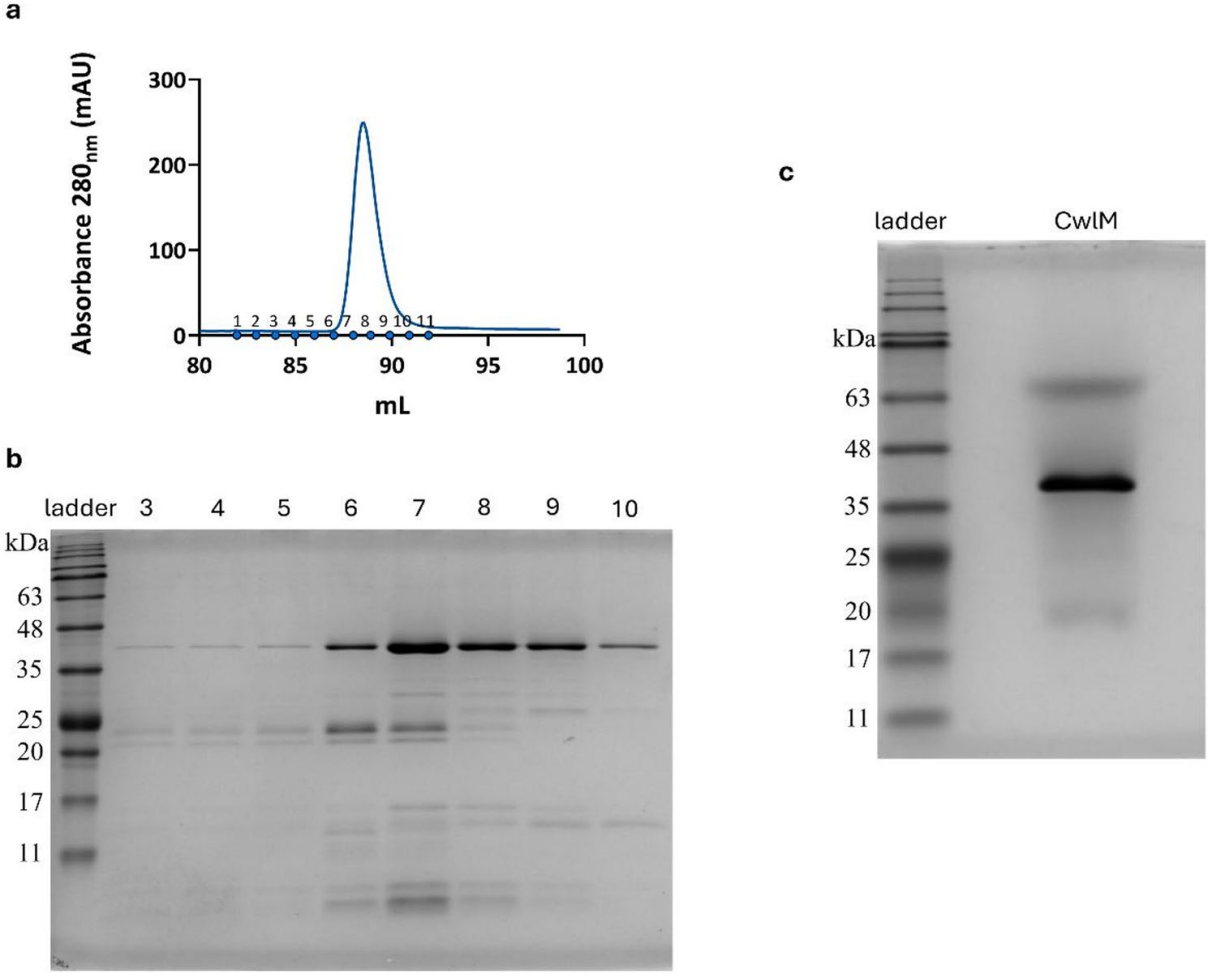
Purification of the CwlM protein.** a** UV absorbance at 280 nm measured by the ÄKTAprime plus system during the elution step of the affinity purification of CwlM_TB_ using a 1 mL HisTrap FF Crude column from an induced filtrate of *E. coli* BL21(DE3):pET-29b:*cwlM*. Fractions 1–11 were collected for analysis. **b** SDS-PAGE gel of the collected fractions of the CwlM protein after purification in the AKTAprime system. Lane order: NZYColour Protein Marker II ladder; fractions 3, 4, 5, 6, 7, 8, 9, and 10. **c** SDS-PAGE of the final purified CwlM protein was performed to confirm protein identity and purity. Lane order: NZYColour Protein Marker II ladder; CwlM protein (predicted size of 43.9 kDa)

Purification of CwlM_TB_ yielded a strong absorbance peak between fractions 7 and 11, with a maximum of about 250 mAµ at 280 nm (Fig. 5). SDS-PAGE analysis confirmed that fractions 6–9 contained most of the concentrated protein, as these fractions exhibited large bands at ~ 43.9 kDa in the gel, matching the expected molecular weight of CwlM_TB_ (Fig. 5). After buffer exchange and imidazole removal, the final purified CwlM_TB_ protein was obtained at a concentration of about 800 µg/µL [40]. SDS-PAGE analysis following dialysis (Fig. 5) revealed several bands: while the inferior bands might indicate a trivial amount of degraded protein, the upper band might represent an aggregate of CwlM with a smaller fragment.

### Characterization of enzymatic activity via zymography and plate assays

**Fig. 6:**
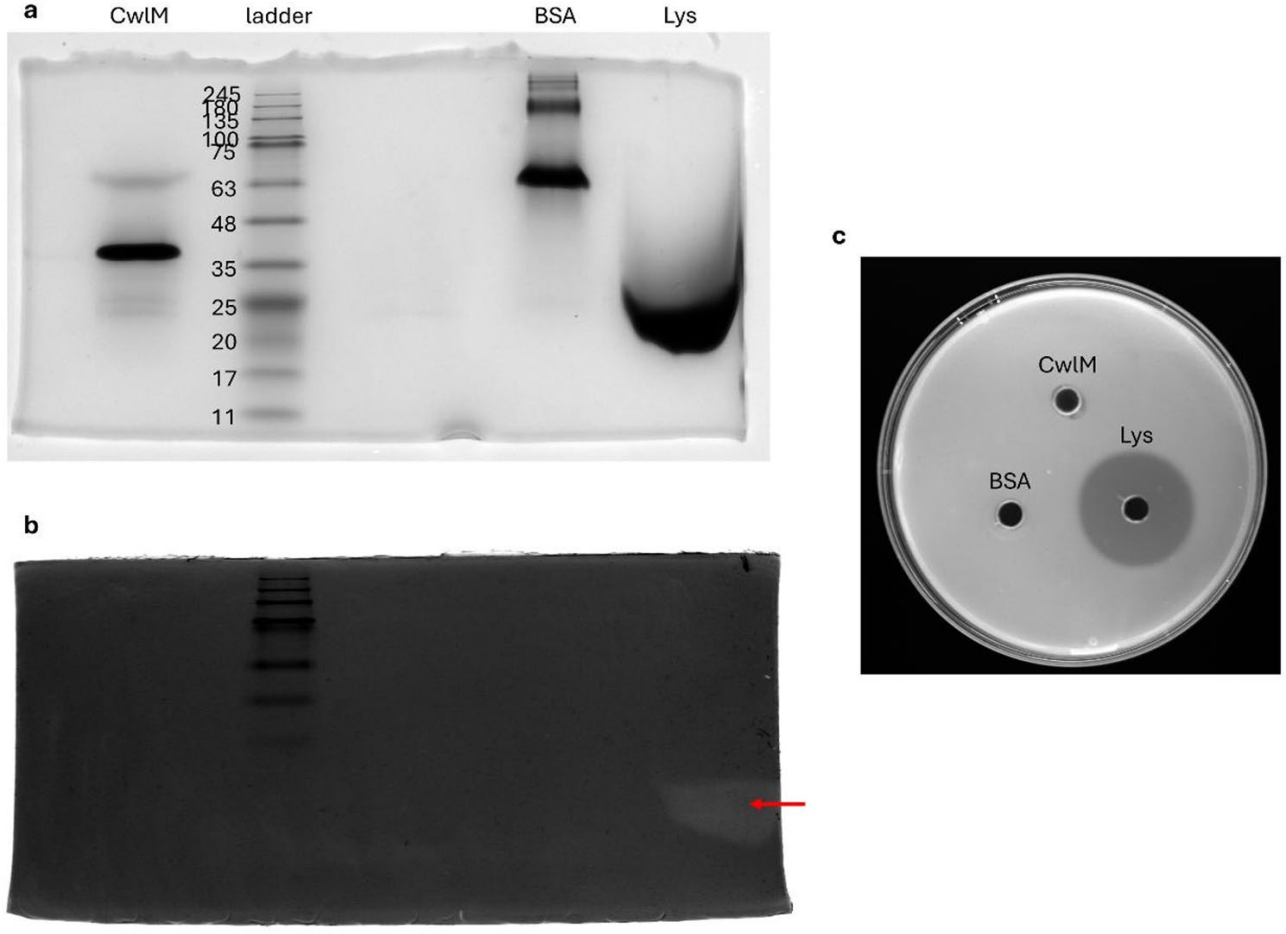
Deciphering the enzymatic activity of CwlM through a zymogram containing 2% of *M. luteus*-derived peptidoglycan.** a** SDS-PAGE analysis of CwlM and two controls (BSA and lysozyme). **b** The zymogram gel shows that the positive control lysozyme can cleave PG from *M. luteus* while the predicted PG hydrolase CwlM cannot. The translucent zone created by lysozyme-mediated PG hydrolysis is indicated with a red arrow. **a**,** b** Lane order of both gels: CwlM; NZYColour Protein Marker II ladder; BSA (negative control); and Lysozyme (Lys – positive control). **c** Plate of *M. luteus* to which the control proteins and CwlM were added. This assay demonstrates the PG-hydrolytic activity of the positive control lysozyme while reiterating the lack of PG-hydrolytic activity of CwlM

To elucidate whether CwlM possesses any PG-hydrolytic activity, a zymogram protocol similar to the one described in [30], was performed using PG from *M. luteus* as substrate (Fig. 6a, b). The zymogram displayed one translucent zone around 14 kDa, consistent with the lytic activity of lysozyme over the PG (positive control - Lys) (Fig. 6b). Nevertheless, the Lys band displays a molecular weight between 17 and 25 kDa, having shifted from its predicted size. This could have occurred because of the low concentration of buffers in the zymogram gel, which may impact protein migration, or due to partial renaturation of the Lys protein resulting from prolonged heat exposure during the assay. On the other hand, no translucent zone was observed with the negative control BSA (66 kDa), as it lacks PG-hydrolytic activity. When compared to lysozyme, CwlM_TB_ does not appear to possess any hydrolase activity against *M. luteus* PG (Fig. 6b) [40]. To further validate these results, a plate assay was performed. While the PG-hydrolytic activity of Lys produced a discernible halo on a *M. luteus* lawn, no halo was observable with CwlM_TB_ (Fig. 6c) [40].

## Discussion

The development of novel antitubercular agents is essential to gain control of DR-TB [2]. The discovery of novel therapeutic targets can be propelled by research into mycobacterial PG biosynthesis enzymes and inhibitors. Although traditionally excluded from DR-TB therapeutic programs, beta-lactams have resurged as promising additions to pipeline drugs, especially when paired with beta-lactamase inhibitors. The WHO now includes two carbapenems in Group C of the medicines recommended for longer MDR-TB therapeutic programs [46]. Recently, we identified several genomic drivers underlying differential beta-lactam susceptibility phenotypes in *Mtb* [29, 47]. Among these, the 4,403,900 snp A > G in *cwlM* was often associated with increased susceptibility to amoxicillin [29]. Therefore, we sought to investigate the role of CwlM in beta-lactam susceptibility and intracellular survival using *M. smegmatis* as a model. Due to previous conflicting information, we also purified recombinant CwlM_TB_ to characterize its function. Our findings, discussed below, suggest that CwlM_SMEG_ contributes to beta-lactam tolerance and intracellular survival, while CwlM_TB_ lacks PG-hydrolytic activity and might act as a PG biosynthesis regulator instead [31].

First, the qRT-PCR results confirmed the successful CRISPRi-mediated knockdown of *cwlM* and the absence of leaky expression phenomena. Notably, the observed repression levels are very mild compared with the 11.1- to 226-fold repression previously reported for target genes in *M. smegmatis* [34], although those levels were attained with twice the induction period used here. Globally, the dCas9_Sth1_-based CRISPRi system effectively inhibited target mRNA inhibition and consequently affected the resultant phenotypes.

Consistent with previous reports, the spotting dilution assays revealed a severe growth defect upon *cwlM* knockdown, confirming its essentiality for mycobacterial survival [30, 48–50]. Notably, even when using a low-strength PAM (PAM14), CRISPRi-mediated silencing of *cwlM* resulted in a near-lethal phenotype, thus underscoring the high vulnerability of *cwlM*, a main criterion for target-based drug discovery [51]. CwlM comprises two PG-binding domains and one MurNAc-LAA domain, with predicted N-acetylmuramoyl-L-alanine amidase activity. Nonetheless, a previous report found that the overexpression of CwlM did not result in lysis, presumably due to the lack of two essential catalytic residues (R199, R270) [31]. Instead, a regulatory function was proposed for CwlM, as phosphorylated CwlM was shown to stimulate the activity of MurA and potentiate PG biosynthesis. As follows, CwlM depletion is likely to deter PG biosynthesis [32], thereby impairing CW integrity, and compromising PG transpeptidation and septum formation. Hence, the regulatory role of CwlM may affect several pathways and justify its essentiality for cell division and survival.

The MIC assays demonstrated that silencing *cwlM* slightly increased susceptibility to beta-lactams, specifically to CTX (+ CLA). Likewise, *cwlM* repression in *Mycobacterium abscessus* also promoted increased susceptibility to beta-lactams [52, 53]. In our experiments, no major susceptibility differences were observed with AMX, possibly because this penicillin is only able to target PBPs [54]. Whereas the addition of CLA did not greatly impact the MICs of CTX and MEM, it provoked a 16-fold reduction in the MIC of AMX, which can be justified by the higher affinity of BlaS for penicillins than for cephalosporins and carbapenems [55]. Overall, MEM was very efficient at inhibiting mycobacterial growth compared to AMX and CTX. Although MEM and CTX inhibit both PBPs and Ldts [56], only MEM can do so efficiently and simultaneously escape the hydrolyzing activity of beta-lactamases [57]. Since the activity of MEM is sufficient to inhibit all transpeptidases and halt PG biosynthesis, the depletion of CwlM did not greatly affect the MIC of MEM. On the other hand, considerable susceptibility differences were observed with CTX (+ CLA), suggesting that CwlM depletion facilitates its activity, likely owing to abnormal cell elongation and uncoordinated synthesis of CW layers [31]. We hypothesize that some PG biosynthesis processes may remain active under *cwlM* repression, facilitating CTX-mediated inhibition of PG cross-linking and further accentuating PG polymerization defects and, consequently, overall PG integrity. Similarly to beta-lactams, the glycopeptide VAN inhibits PG transpeptidation by irreversibly binding the D-Ala-D-Ala dipeptide [58]; however, the induced *cwlM* KDM did not exhibit increased susceptibility to VAN, possibly because the D-Ala-D-Ala moiety is protected by the lipid-rich CW of mycobacteria. In this way, VAN might only inhibit PG cross-linking when combined with first-line agents EMB and INH [40, 58].

The spotting dilution assays in the presence of varying concentrations of MEM and CTX showed that CwlM depletion resulted in subtle susceptibility increases to both beta-lactams. These observations do not fully replicate but are consistent with the trends observed in the MIC assays. The disk diffusion results suggest that CwlM depletion may slightly increase susceptibility to MEM, further emphasizing its high potency against mycobacteria. A similar trend was observed for AMX + CLA, yet without statistical significance.

Altogether, the susceptibility assays suggest that CwlM contributes to beta-lactam tolerance, particularly to CTX and MEM. Accordingly, a recent study showed that *cwlM* depletion in *M. smegmatis* increased susceptibility to β-lactams, namely to cefoxitin and imipenem, due to increased CW permeability [59]. Although we recognize that the observed susceptibility changes may partially reflect impaired growth, the antibiotic-specific effects indicate that altered CW integrity and permeability following *cwlM* repression may also influence antibiotic diffusion and contribute to these phenotypes. While not directly tested here, this possibility should be addressed in future studies, including CW permeability or ultrastructural analyses.

The cytosolic NOD-like receptors (NLRs), NOD1 and NOD2, recognize molecules containing D-Glu-*m*-DAP and PG–derived muramyl dipeptide (MDP), respectively [7, 8]. The intracellular survival assays showed that the depletion of CwlM resulted in a faster clearance of intracellular mycobacteria, possibly due to impaired PG integrity and increased susceptibility to macrophage-mediated clearance. While *M. smegmatis* is widely used to study macrophage-mycobacteria interactions, it is a primarily saprophytic bacterium that is rapidly cleared by macrophages; therefore, the substantial survival drop of the induced *cwlM* KDM shortly after internalization may simultaneously reflect its intrinsic fragility and the host environment. Phosphorylated CwlM is known to stimulate PG biosynthesis by 30-fold [31], thus promoting the activity of mycobacterial PG-modifying enzymes. It is inferable that *cwlM* repression impairs PG biosynthesis, leading to deficient PG polymerization, which could facilitate recognition of PG fragments by host macrophages. The depletion of CwlM was induced 24 h before infection, and although starting OD_600_ values were comparable among the study strains, the induced *cwlM* KDM population may have already been compromised and highly susceptible to additional stresses at the time of infection. This underscores the dual role of CwlM: not only is it essential for bacterial viability under basal conditions, but it also contributes to intracellular survival, perhaps by promoting efficient PG biosynthesis.

The CwlM protein is annotated as a PG hydrolase that cleaves an amide bond between the glycan moiety (Mur*N*Ac) and the peptide moiety (L-alanine) [30]. However, several studies showed that CwlM lacks enzymatic activity, rather serving a regulatory function [31, 32]. Here, we successfully purified the His_6_-tagged CwlM_TB_ and attempted to determine whether CwlM_TB_ possesses amidase activity through zymogram assays. PG from *M. luteus* is frequently used for the detection of amidase activity against the CW of gram-positive bacteria [30, 60, 61]. In this case, the PG from *M. luteus* was used as a substrate because it enabled the assessment of PG-hydrolytic activity without the limitation provided by the external layers of the mAGP complex. Collectively, the zymogram and plate assays showed that CwlM_TB_ lacks PG-hydrolytic activity. Although our results contradict the findings of a previous study [30], they are concordant with the recent hypothesis proposing that CwlM plays a regulatory role in PG biosynthesis [31, 32].

Building on the role of CwlM in PG biosynthesis, a recent study showed that the activity of PknB, the STPK for which CwlM is a major substrate, is stimulated by the depletion of D-*i*Glu amidation [14]. Accordingly, the depletion of D-*i*Glu amidation leads to perturbed PG cross-linking, hyperactivation of PknB, and ultimately reduced *de novo* PG biosynthesis. Aligned with this, we recently demonstrated that the depletion of D-*i*Glu amidation increases susceptibility to beta-lactams [6], likely due to downregulated Ldt activity and increased dependence on PBP activity, which is easily inhibited by these antibiotics [14]. The hyperactivation of PknB stimulates the phosphorylation of its substrates (e.g., CwlM, MurA, MurJ, and others) [14, 32, 62]. In this case, phosphorylated CwlM binds to FhaA, stabilizing PbpA activity while inhibiting MurJ activity. Hence, lipid II accumulates in the cytoplasm, resulting in reduced *de novo* PG biosynthesis [32]. Should the activity of PknB remain unregulated, this will eventually cause bacteriolysis.

Our work is not deprived of limitations. First, the CRISPRi-mediated repression of *cwlM* employed a medium-to-low strength PAM (PAM 14). Although the obtained levels of repression of *cwlM* were modest (about 3-fold), these were sufficient to cause pronounced growth defects. While our study aimed to investigate the role of *cwlM* in antibiotic susceptibility and intracellular survival, the observed growth impairment emphasizes the significance of CwlM for mycobacterial fitness. In fact, this near-lethal phenotype complicates the interpretation of drug susceptibility and intracellular survival data, as effects may primarily reflect *cwlM* essentiality. Nonetheless, the observed growth and viability defects indicate that *cwlM* is both essential and highly vulnerable, highlighting its potential as a therapeutic target. Future studies should perform a dose-response assessment to find sublethal concentrations of ATc that lead to efficient gene knockdown without causing extensive growth deficits. Although we did not perform experimental verification of the phosphorylation state of the purified protein, CwlM_TB_ was likely non-phosphorylated considering that *E. coli* does not express PknB_TB_. Owing to its rapid growth, genetic homology, and conservation of core metabolic pathways, *M. smegmatis* remains a widely used surrogate model for *Mtb*, particularly in exploratory studies such as this one [63, 64]. Nevertheless, we acknowledge that differences in cell envelope composition, beta-lactamase activity, and virulence may limit direct extrapolation to *Mtb*, and additional assays are necessary to confirm the applicability of our findings in the pathogen. In addition, future studies should validate these findings in vivo.

## Conclusions

In conclusion, our findings suggest that CwlM may contribute to beta-lactam tolerance and intracellular survival, thereby limiting antimicrobial efficacy. The essentiality and high vulnerability the *cwlM* gene were confirmed by the pronounced growth defects observed upon its repression, thus emphasizing the potential of CwlM as a therapeutic target. In addition to this, we confirmed that purified CwlM_TB_ lacks PG-hydrolytic activity and suggested it may rather participate in the regulation of PG biosynthesis, as was previously hypothesized [31]. Overall, targeting CwlM-regulated processes could enhance antibiotic sensitivity and potentially inform strategies to shorten treatment regimens for DR-TB.

## Supplementary Information

Additional file 1.

## Data Availability

All data supporting the findings of this study are available within the paper and its Supplementary Information. No new datasets requiring public deposition were generated. Further inquiries can be directed to the corresponding author and are available on reasonable request.
