# Supplementary material for: Effects of CwlM, a peptidoglycan synthesis regulator, on beta-lactam tolerance and host-pathogen interactions
Source: BMC Microbiol. 2025 Dec 7;26:21. doi: 10.1186/s12866-025-04548-6 (PMC12797351; doi:10.1186/s12866-025-04548-6)
Supplement: Supplementary file 1 — Additional file 1. [file 12866_2025_4548_MOESM1_ESM.pdf]

Supplementary Material

1 Supplementary Figures and Tables

1.1 Supplementary Figures

AGATGAGATGGTCAGATTAACTGGCTGACGGAATTTATGCTTCTCCGACCATCAAGCATTTATCCGTACTCCTG
ATGATGCATGGTTACTCACCACTGCGATCCCCGGGAAAACAGCATTCCAGGTATTAGAAGAATATCCTGATTCA
GGTGAAAATATTGTTGATGCGCTGGCAGTGTTCCTGCGCCGGTTGCATTCGATTCCTGTTTGTAATTGTCCTTTT
AACAGCGATCGCGTATTTCGCCTCGCTCAGGCGCAATCACGAATGAATAACGGTTTGGTTGATGCGAGTGATTC
TGATGACGAGCGTAATGGCTGGCCTGTTGAACAAGTCTGGAAAGAAATGCATAATCTTTTGCCATTCTCACCGG
ATTCAGTCGTCACTCATGGTGATTTCTCACTTGATAACCTTATTTTTGACGAGGGGAAATTAATAGGTTGTATTG
ATGTTGGACGAGTCGGAATCGCAGACCGATACCAGGATCTTGCCATCCTATGGAACTGCCTCGGTGAGTTTTCT
CCTTCATTACAGAAACGGCTTTTTCAAAAATATGGTATTGATAATCCTGATATGAATAAATTGCAGTTTCATTTG
ATGCTCGATGAGTTTTTCTAATCAGAATTGGTTAATTGGTTGTAACACTGGCAGAGCATTACGCTGACTTGACG
GGACGGCGGCTTTGTTGAATAAATCGAACTTTTGCTGAGTTGAAGGATCAGATCACGCATCTTCCCGACAACGC
AGACCGTTCCGTGGCAAAGCAAAAGTTCAAAATCACCAACTGGTCCACCTACAACAAAGCTCTCACCAACCGTG
GCTCCCTCACGATATCAATAAACGAAAGGCTCAGTCGAAAGACTGGGCCTTTCGTTTATCTGTTGTTTGAAAAA
AAAAAGCGCCGCAACTGCGGCGCTTTTTTTTTTGAATTCTCTGACCAGGGAAAATAGCCCTCTGACCTGGGGAT
TTGCGATCCCTATCAGTGATAGATATAATCTGGGAACCCGCCGGTGACGCGTGAGCGTTTTTGTACTCGAAAGA
AGCTACAAAGATAAGGCTTCATGCCGAAATCAACACCCTGTCATTTTATGGCAGGGTGTTTTTTTTTTGTCGACT
TGGGGACCCTAGAGGTCCCCTTTTTTTTTTGA

Figure S1 - Sequencing results (5' – 3') of PLJR962 with the sgRNA targeting the gene of interest (*cwlM*) in *M. smegmatis*. The -10 site is underlined in yellow, the insert or sgRNA is underlined in green and the dCas9 handle is painted in grey.

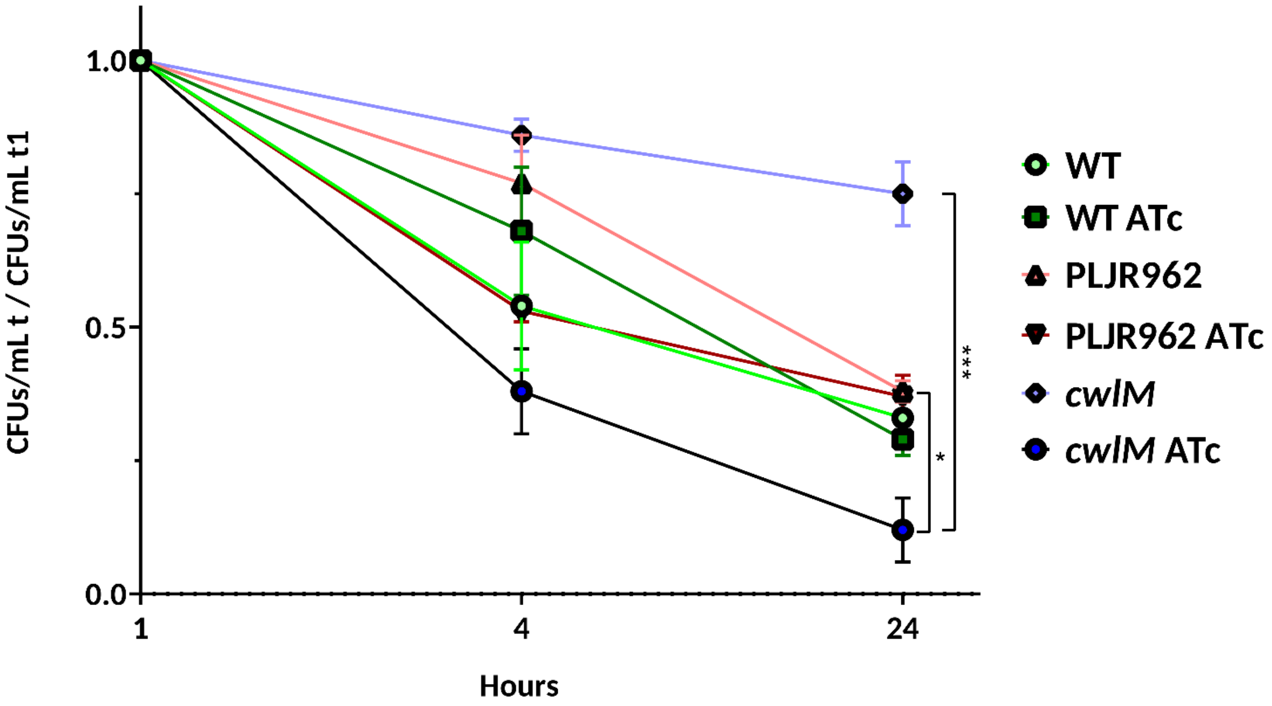

Figure S2 – Ratio of bacterial survival (in CFUs/mL) in infected and disrupted PMA-differentiated THP-1 macrophages at 4 h and 24 h post-infection, compared to intracellular survival at 1 hour (t1) (n=3). Comparative effect with and without 100 ng/mL of inducer (ATc). Error bars represent the standard error of the mean (SEM). Multiple comparisons were calculated using one-way ANOVA (* $P < 0.05$; *** $P < 0.001$).

a. pET-29b:*cwlM* with primer Fwd T7:

GGCGTACATTCCCTCTAGAATAATTTTGTTTAACTTTAAGAAGGAGATATACATATGCCGAGTCCGCGCCGCGAAGACG
GCGATGCGCTGCGCTGTGGCGACCGCAGTGCGGCCGTCACCGAGATCCGGGCTGCGCTGACCGCGTTAGGGATGCTG
GATCATCAGGAAGAAGACCTGACGACGGGCCGTAACGTCGCCCTTGAGTTGTTCGACGCGCAGCTCGACCAGGCGGTC
CGTGCCTTCCAACAGCATCGCGGCCTGCTGGTGGACGGCATCGTCGGTGAGGCCACCTACCGCGCGTTGAAAGAAGCC
TCCTACCGGCTCGGGGCCCGCACGCTGTACCACCAATTCGGCGCCCCGCTCTACGGGGACGACGTCGCTACACTGCAGG
CCCGGCTGCAGGATCTTGGTTTCTACACCGGGCTGGTCGACGGTCATTTCGGGTTGCAGACCCACAATGCGTTGATGTC
CTATCAGCGTGAGTACGGACTTGCCGCAGACGGTATCTGCGGCCCAGAAACGTTGCGCTCCTTGTACTTTCTAAGTTCG
CGAGTCAGCGGTGGCTCGCCACATGCGATTCGCGAAGAAGAGCTGGTCCGCAGCTCGGGGCCGAAGCTGTCTGGCAA
ACGGATCATCATTGATCCCGGTCGCGGCGGCGTGGACCACGGACTTATCGCGCAAGGTCCGGCTGGGCCCATCAGCGA
AGCAGACTTGTTGTGGGACTTGGCAAGTCGGCTCGAAGGACGGATGGCAGCTATCGGTATGGAGACCCACCTGTCCCG
TCCGACCAACCGTAGTCCGTCCGACGCAGAGCGTGCCGCCACCGCCAACGCCGTTGGCGCAGACCTGATGATCAGCCT
GCGCTGCGAGACCCAGACCAGTCTCGCGGCCAACGGCGTGGCTTCCTTTCACTTCGGCAACTCGCACGGCTCGGTGTCT
ACCATCGGCCGCAATCTTGCCGATTTCATTCAACGAGAAGTGGTGGCGCGCACCGGTTTACGGGATTGCCGTGTGCATG
GTCGAACGTGGGATCTGTTGCGGCTGACCAGGATGCCGACCGTTCAGGTCGATATCGGCTACATCACCAACCCCCACGA
TCGTGGGATGCTGGTCTCAACGCAGAACGC

b. pET-29b:*cwlM* with primer Rv T7:

AGCCTCGCTTCTTTCGGGCTTTGTTAGCAGCCGGATCTCAGTGGTGGTGGTGGTGGTGCTCGAGAGAACCGCCGAGTCT
ACCCGCTCGCTCGACAGACAGTTCGTGGGCCAGCAACTCGGCGAAAGTGAATGTGCCGGTGGGCCGATCGTTCTTGCCT
AACAGATACAGCCGTTTGACCGCGGCGAGAATGCCTTCGGCGATGGCATCGCGCGTCTGCGTTGAGACCAGCATCCCAC
GATCGTGGGGGTTGGTGATGTAGCCGATATCGACCTGAACGGTCGGCATCCTGGTCAGCCGCAACAGATCCCACGTTCG
ACCATGCACACGGCAATCCCGTAAACCGGTGCGCGCCACCACTTCTCGTTGAATGAAATCGGCAAGATTGCGGCCGATG
GTAGACACCGAGCCGTGCGAGTTGCCGAAGTGAAAGGAAGCCACGCCGTTGGCCGCGAGACTGGTCTGGGTCTCGCAG
CGCAGGCTGATCATCAGGTCTGCGCCAACGGCGTTGGCGGTGGCGGCACGCTCTGCGTCGGACGGACTACGGTTGGTC
GGACGGGACAGGTGGGTCTCCATACCGATAGCTGCCATCCGTCCTTCGAGCCGACTTGCCAAGTCCCACAACAAGTCTG
CTTCGCTGATGGGCCCAGCCGGACCTTGCGCGATAAGTCCGTGGTCCACGCCGCCGCGACCGGGATCAATGATGATCCG
TTTGCCAGACAGCTTCGGCCCCGAGCTGCGGACCAGCTCTTCTTCGCGAATCGCATGTGGCGAGCCACCGCTGACTCGC
GAACTTAGAAAGTACAAGGAGCGCAACGTTTCTGGGCCGCAGATACCGTCTGCGGCAAGTCCGTACTCACGCTGATAGG
ACATCAACGCATTGTGGGTCTGCAACCCGAAATGACCGTCGACCAGCCCGGTGTAGAAACCAAGATCCTGCAGCCGGGC
CTGCAGTGTAGCGACGTCGTCCCCGTAGAGCGGGGCGCCGAATTGGTGGTACAGCGTGCGGGCCCCGAGCCGGTAGG
AGGCTTCTTTCAACGCGCGGTAGGTGGCCTCACCGACGATGCCGTCCACCAGCAGGCCGCGATGCTGTTGGAAGGCACG
GACCGCCTGGTCAAGCTGCGCGTCAAAAAC

Figure S3 - Sequencing results (5' – 3') of pET29b with the T7 promoter a) forward and terminator b) reverse primers. Fwd – forward, Rv – reverse. The insert is underlined in green; the histidine tail is underlined in purple; and the XhoI and NdeI restriction sites are underlined in grey and light blue, respectively.

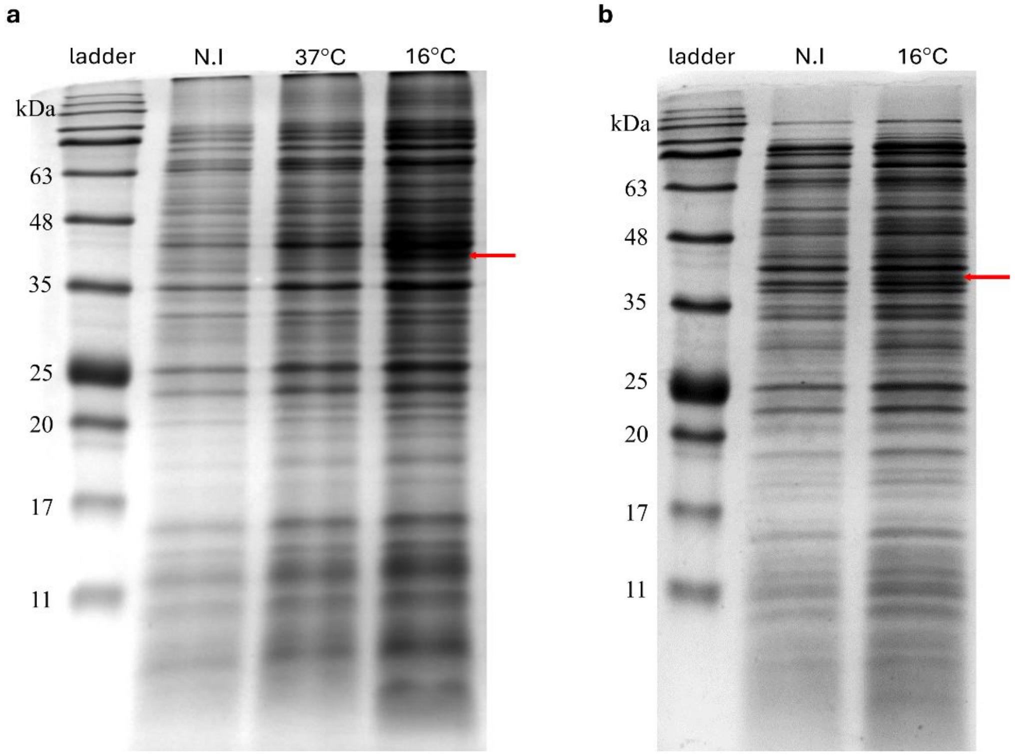

Figure S4 - SDS polyacrylamide gel analysis of cell lysate supernatants for the optimization of the expression and induction conditions for the purification of the CwlM protein. Protein separation is based on size and 10% acrylamide gels were used. The size of CwlM is predicted to be about 43.9 kDa. The red arrows indicate the presence of the protein of interest. **(a)** SDS-PAGE of the supernatants of non-induced and induced cell lysates of *E. coli* BL21 (DE3) expressing CwlM in different conditions. Lane order: NZYColour Protein Marker II ladder; non-induced (N.I) *E. coli* BL21(DE3):pET-29b:*cwlM*; *E. coli* BL21(DE3):pET-29b:*cwlM* induced at 37°C for 3 h; *E. coli* BL21(DE3):pET-29b:*cwlM* induced at 16°C overnight. **(b)** SDS-PAGE of the supernatant of the non-induced and induced cell lysates of *E. coli* BL21 (DE3) expressing CwlM at 16°C, overnight**.** Lane order: ladder NZYColour Protein Marker II ladder; non-induced (N.I) *E. coli* BL21(DE3):pET-29b:*cwlM*; and *E. coli* BL21(DE3):pET-29b:*cwlM* induced at 16°C overnight.

1.2 Supplementary Tables

Table S1 - Average of three independent experiments measuring the optical density (at 600 nm) **of the study strains** (*M. smegmatis* WT, PLJR962 and the *cwlM* knockdown mutant, grown with and without 100 ng/mL of ATc) **immediately before infection.**

**Optical density (600 nm) immediately before infection**	
M. smegmatis	
WT	0.57
WT ATc	0.50
PLJR962	0.41
PLJR962 ATc	0.43
cwlM	0.34
cwlM ATc	0.41
